# Supplementary material for: First Observation of Embryonic Development and Paralarvae of Amphioctopus kagoshimensis
Source: Animals (Basel). 2025 Nov 10;15(22):3249. doi: 10.3390/ani15223249 (PMC12649247; doi:10.3390/ani15223249)
Supplement: Supplementary file 1 [file animals-15-03249-s001.zip › Supplementary Code S1.pdf]

```
# ==================================================
# Supplementary Code S1 — Growth analyses for A. kagoshimensis
# R version: 4.2.2
# ==================================================

# ---- 0) Packages (no installation here; use base when possible)
suppressPackageStartupMessages({
  ok_car <- requireNamespace("car", quietly = TRUE)
  library(ggplot2)
})

# ---- 1) Input data (stage means; mm)
dat <- data.frame(
  days = c(1, 3, 5, 7, 10, 15, 20, 30),
  TL   = c(3.44, 3.66, 3.85, 3.93, 4.06, 4.30, 4.63, 5.02),
  ML    = c(1.58, 1.60, 1.65, 1.75, 1.81, 1.92, 1.97, 2.03),
  AL   = c(1.35, 1.45, 1.52, 1.58, 1.70, 1.85, 1.95, 1.90)  # note 30 d slight dip
)

# ---- 2) SGR (interval and overall)
calc_SGR <- function(len, t) (log(len[-1]) - log(len[-length(len)])) / (t[-1] - t[-length(t)]) *
100
intervals <- paste0(head(dat$days, -1), "-", tail(dat$days, -1))
```

```r
SGR_tbl <- rbind(
  data.frame(Trait="TL", Interval=intervals, SGR=round(calc_SGR(dat$TL, dat$days), 3)),
  data.frame(Trait="ML", Interval=intervals, SGR=round(calc_SGR(dat$ML, dat$days), 3)),
  data.frame(Trait="AL", Interval=intervals, SGR=round(calc_SGR(dat$AL, dat$days), 3))
)

avg_SGR <- function(len, t) (log(tail(len,1)) - log(head(len,1))) / (tail(t,1)-head(t,1)) * 100
overall_SGR <- data.frame(
  Trait = c("TL","ML","AL"),
  Avg_SGR = round(c(avg_SGR(dat$TL, dat$days),
                    avg_SGR(dat$ML, dat$days),
                    avg_SGR(dat$AL, dat$days)), 3)
)

# ---- 3) Time-based models
fit_ML_time <- lm(ML ~ days, data = dat)
fit_AL_time <- lm(AL ~ days, data = dat)

# Exponential TL = a * exp(b * t)
start_exp <- list(a = min(dat$TL)*0.95, b = 0.02)
fit_TL_exp <- nls(TL ~ a * exp(b * days), data = dat, start = start_exp, control =
nls.control(maxiter = 500))

# R^2 for nls (pseudo-R^2)
```

```
pseudo_r2 <- function(obs, fit) {
  rss <- sum((obs - fit)^2); tss <- sum((obs - mean(obs))^2); 1 - rss/tss
}

r2_ML_time <- summary(fit_ML_time)$r.squared
r2_AL_time <- summary(fit_AL_time)$r.squared
r2_TL_time <- pseudo_r2(dat$TL, fitted(fit_TL_exp))

# ---- 4) Multiple regression TL ~ ML + AL with VIF
fit_multi <- lm(TL ~ ML + AL, data = dat)

if (ok_car) {
  VIF <- as.data.frame(car::vif(fit_multi))
  VIF$Predictor <- rownames(VIF); rownames(VIF) <- NULL
  names(VIF)[1] <- "VIF"
} else {
  # Base VIF fallback: regress each predictor on others
  base_vif <- function(X) {
    out <- numeric(ncol(X)); names(out) <- colnames(X)
    for (j in seq_len(ncol(X))) {
      y <- X[,j]; Xj <- X[,-j, drop=FALSE]
      r2 <- summary(lm(y ~ ., data = as.data.frame(Xj)))$r.squared
      out[j] <- 1/(1 - r2)
    }
```

```
    out
  }
  X <- model.matrix(~ ML + AL, data = dat)[, -1, drop=FALSE]
  VIF <- data.frame(Predictor = colnames(X), VIF = round(base_vif(X), 3))
}

# ---- 5) Plots (with R^2)
theme_clean <- theme_bw(base_size = 13) + theme(plot.title = element_text(hjust=0.5),
                                                 plot.subtitle = element_text(hjust=0.5))

# ML vs time
p_ML_time <- ggplot(dat, aes(days, ML)) +
  geom_point() +
  geom_smooth(method = "lm", se = TRUE, linewidth = 1) +
  labs(x="Days post-hatching", y="Mantle length (mm)",
       title="ML vs time", subtitle = paste0("R² = ", round(r2_ML_time, 3))) +
  theme_clean

# AL vs time
p_AL_time <- ggplot(dat, aes(days, AL)) +
  geom_point() +
  geom_smooth(method = "lm", se = TRUE, linewidth = 1) +
  labs(x="Days post-hatching", y="Arm length (mm)",
       title="AL vs time", subtitle = paste0("R² = ", round(r2_AL_time, 3))) +
```

```
  theme_clean

# TL vs time (exponential curve)
t_new <- data.frame(days = seq(min(dat$days), max(dat$days), length.out = 200))
pred_TL <- predict(fit_TL_exp, newdata = t_new)
p_TL_time <- ggplot() +
  geom_point(data = dat, aes(days, TL)) +
  geom_line(aes(t_new$days, pred_TL), linewidth = 1) +
  labs(x="Days post-hatching", y="Total length (mm)",
       title="TL vs time (exponential fit)",
       subtitle = paste0("pseudo-R² = ", round(r2_TL_time, 3))) +
  theme_clean

# Optional: TL vs ML / TL vs AL (for visualization only)
p_TL_ML <- ggplot(dat, aes(ML, TL)) +
  geom_point() +
  geom_smooth(method = "lm", se = TRUE, linewidth = 1) +
  labs(x="Mantle length (mm)", y="Total length (mm)",
       title="TL vs ML (visualization)") + theme_clean

p_TL_AL <- ggplot(dat, aes(AL, TL)) +
  geom_point() +
  geom_smooth(method = "lm", se = TRUE, linewidth = 1) +
  labs(x="Arm length (mm)", y="Total length (mm)",
```

```r
    title="TL vs AL (visualization)") + theme_clean

# ---- 6) Export outputs
dir.create("supp_outputs", showWarnings = FALSE)
ggsave("supp_outputs/Fig4A_TL_time.png", p_TL_time, width=6, height=4.2, dpi=300)
ggsave("supp_outputs/Fig4B_ML_time.png", p_ML_time, width=6, height=4.2, dpi=300)
ggsave("supp_outputs/Fig4C_AL_time.png", p_AL_time, width=6, height=4.2, dpi=300)
ggsave("supp_outputs/FigS_TL_vs_ML.png", p_TL_ML, width=6, height=4.2, dpi=300)
ggsave("supp_outputs/FigS_TL_vs_AL.png", p_TL_AL, width=6, height=4.2, dpi=300)

write.csv(SGR_tbl, file = "supp_outputs/SGR_by_interval.csv", row.names = FALSE)
write.csv(overall_SGR, file = "supp_outputs/SGR_overall.csv", row.names = FALSE)

# Model metrics table
metrics <- data.frame(
  Model = c("ML ~ time (lm)", "AL ~ time (lm)", "TL ~ time (exp nls)"),
  R2_or_pseudoR2 = round(c(r2_ML_time, r2_AL_time, r2_TL_time), 4),
  AIC = c(AIC(fit_ML_time), AIC(fit_AL_time), AIC(fit_TL_exp))
)
write.csv(metrics, file = "supp_outputs/model_metrics_AIC_R2.csv", row.names = FALSE)

# VIF export
write.csv(VIF, file = "supp_outputs/multiple_regression_VIF.csv", row.names = FALSE)
```

```
# Print concise summary to console
cat("\n=== Interval SGR (%, by trait) ===\n"); print(SGR_tbl)
cat("\n=== Overall SGR (%, 1–30 dph) ===\n"); print(overall_SGR)
cat("\n=== Multiple regression TL ~ ML + AL ===\n"); print(summary(fit_multi))
cat("\n=== VIF ===\n"); print(VIF)
cat("\nOutputs saved under ./supp_outputs\n")
```
