# Supplementary material for: First Observation of Embryonic Development and Paralarvae of Amphioctopus kagoshimensis
Source: Animals (Basel). 2025 Nov 10;15(22):3249. doi: 10.3390/ani15223249 (PMC12649247; doi:10.3390/ani15223249)
Supplement: Supplementary file 1 [file animals-15-03249-s001.zip › Supplementary figures.pdf]

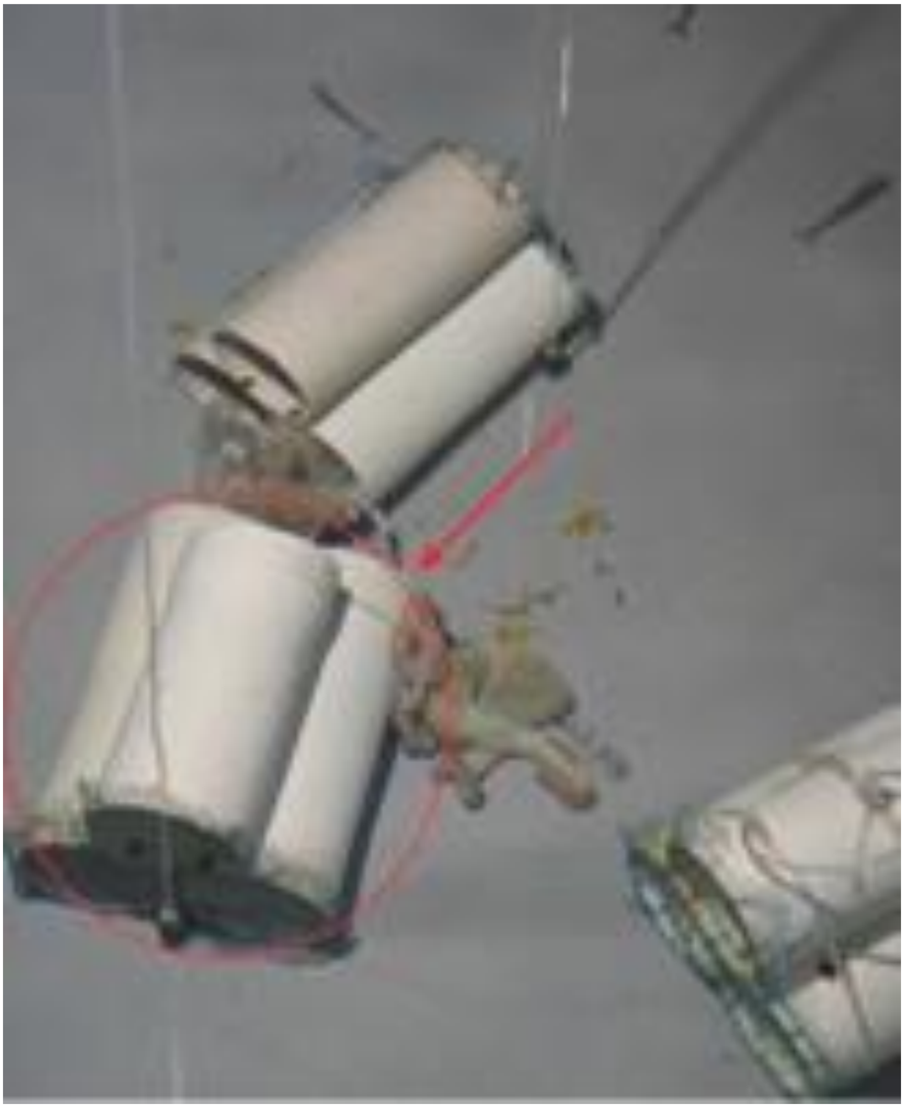

Supplementary Figure S1. Custom-designed refuge structure used for egg incubation of *Amphioctopus kagoshimensis*. The device consisted of three interconnected polyvinyl chloride (PVC) buckets (15 cm × 40 cm each) bound together with mesh-covered openings to allow water exchange while preventing egg loss. Adult females deposited and guarded egg masses within the internal chambers under controlled laboratory conditions.

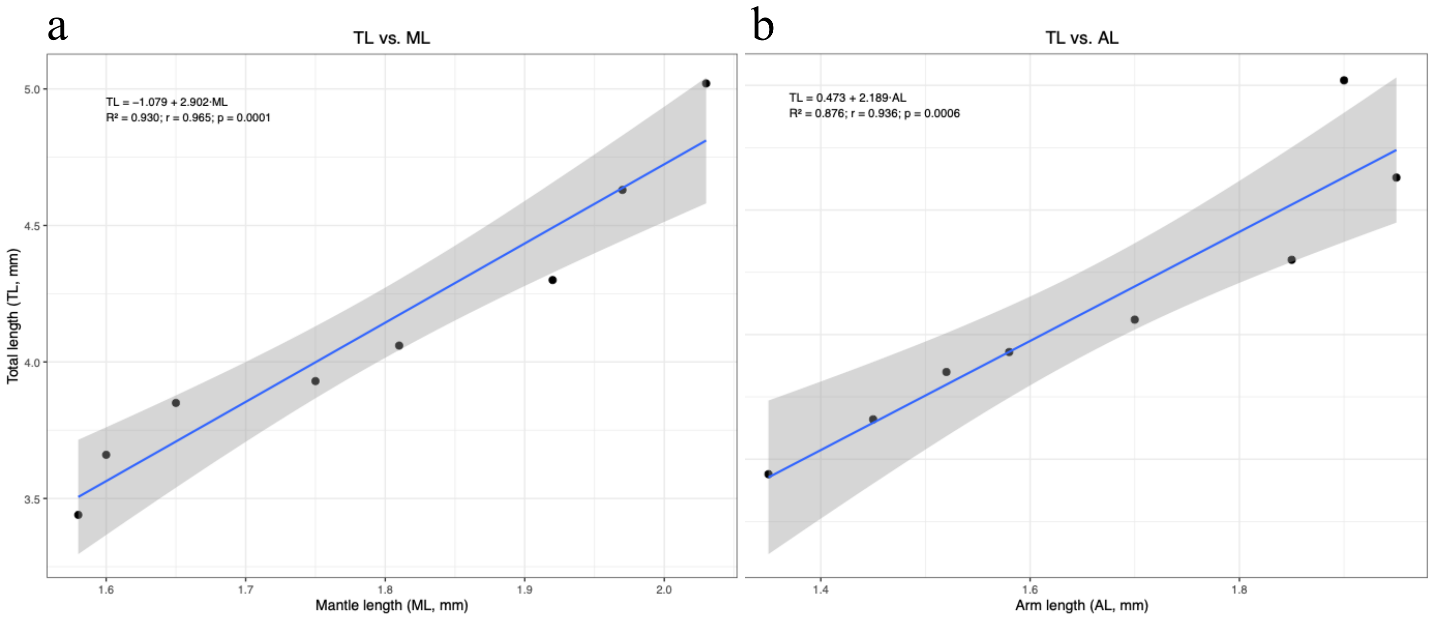

Supplementary Figure S2. Relationships between total length (TL) and mantle length (ML) (a), and between total length (TL) and arm length (AL) (b) of *Amphioctopus kagoshimensis* paralarvae during 1–30 days post-hatching (dph). Each plot shows linear regression lines with 95% confidence intervals. Analyses were performed in R (v4.2.2); regression equations and R^2 values are indicated on each panel.
